# Feasibility and Safety of Cervical Biopsy Sampling for Mucosal Immune Studies in Female Sex Workers from Nairobi, Kenya

**DOI:** 10.1371/journal.pone.0047570

**Published:** 2012-10-15

**Authors:** Klara Hasselrot, Juliana Cheruiyot, Joshua Kimani, Terry B. Ball, Rupert Kaul, Taha Hirbod

**Affiliations:** 1 Department of Medicine, Unit of Infectious Diseases, Center for Molecular Medicine, Karolinska University Hospital/Karolinska Institutet, Stockholm, Sweden; 2 Department of Medical Microbiology, University of Nairobi, Nairobi, Kenya; 3 Departments of Immunology and Medical Microbiology, University of Manitoba, Winnipeg, Manitoba, Canada; 4 National HIV and Retrovirology Laboratories, Public health Agency of Canada, Ottawa, Ontario, Canada; 5 Departments of Medicine and Immunology, University of Toronto, Ontario, Canada; Rush University, United States of America

## Abstract

**Background:**

There is an urgent need to improve our understanding of the mucosal immuno-pathogenesis of HIV acquisition in the female genital tract, particularly in high-risk women such as female sex workers (FSWs). Cervical biopsy samples offer technical advantages over cytobrush sampling, but there are concerns that this might increase HIV acquisition, particularly if healing is slow and/or women do not abstain from sex during healing.

**Methodology/Principal Findings:**

Cervical biopsy samples and cervico-vaginal swabs for co-infection diagnostics, prostate specific antigen (PSA) and immune studies were collected from 59 women, including HIV seropositive and HIV-exposed seronegative (HESN) FSWs as well as lower risk women from Nairobi, Kenya. A clinical-demographic questionnaire was administered and women were instructed to avoid sexual intercourse, douching and the insertion of tampons for 14 days. All participants underwent a repeat exam to assess healing within the 14 days, and had HIV diagnostics at six months. Cervical sampling was well tolerated, and 82% of participants had healed macroscopically by 5 days. Both self-report and PSA screening suggested high levels of compliance with pre- and post-procedure abstinence. Delayed healing was associated with vulvovaginal candidiasis (VVC) and HESN status. At six-month follow up all low-risk and HESN participants remained HIV seronegative.

**Conclusion:**

Cervical biopsy sampling is a safe and well-tolerated method to obtain cervical biopsies in this context, particularly if participants with VVC are excluded. As healing could be delayed up to 11 days, it is important to support (both financially and with rigorous counseling) a period of post-procedure abstinence to minimize HIV risk.

## Introduction

While the human immunodeficiency virus (HIV) is generally acquired across the mucosal lining of the genital tract or rectum during sex, occasional individuals appear to be relatively resistant to HIV acquisition despite repeated mucosal exposure. Defining the mucosal immune correlates of protection in these subjects could provide important clues for the HIV vaccine and microbicide fields [1]. HIV exposed seronegative individuals (HESN) have been identified in many areas of the world [2], with perhaps the best-defined cohort being a subset of female sex workers (FSW) from Nairobi, Kenya [3].

Immune studies in HESN cohorts initially focused on blood-derived lymphocytes, but later studies emphasized the importance of cervicovaginal samples when investigating possible correlates of protection against HIV [4], [5], [6]. However, the traditional cervicovaginal sampling techniques of lavage and cytobrush are constrained in their ability to define mucosal biology at the intraepithelial or submucosal level. When performed, genital histology studies on HIV uninfected subjects are often restricted to tissues obtained from surgical specimens [7], [8] or from animal models [9]. Previously our group has investigated cervical biopsy specimens from women at a theoretically high risk of acquiring HIV [10]; however, to our knowledge immune studies using tissues from HESN individuals have not been performed.

HESN individuals are generally under intense HIV infection pressure, and so one reason for the lack of mucosal biopsy studies is concern regarding the potential for enhanced HIV susceptibility after cervical/rectal biopsy. However, if such studies could be performed safely according to a well-controlled protocol, then they might provide the field with urgently needed information regarding the mucosal immune correlates of HIV protection. Therefore, the objective of this study was to assess the acceptability and feasibility of obtaining cervical biopsies by using a well tolerated methodology in a cohort of female sex workers with multiple clients. Since HIV is considered endemic in this area it was important to reach a high compliance with the requested period of post-procedure sexual abstinence to minimize HIV exposure.

## Materials and Methods

### Ethics Statement

The study was reviewed and approved by the regional ethical boards at Kenyatta National Hospital, Nairobi, Kenya; the Karolinska Institutet, Stockholm, Sweden; and the University of Manitoba, Winnipeg, Canada. Written informed consent was obtained from all study participants. All ethical committees approved the consent procedure.

### Study Participants

HIV-uninfected and infected FSW participants were recruited at the Majengo Sex Worker Clinic [3] and HIV-uninfected lower risk controls were recruited at a Maternal Health Clinic based at the Pumwani Maternity Hospital [11]. Inclusion criteria were: (1) age >18 years; (2) uterus and cervix present; (3) willingness to undergo pelvic exams and ectocervical biopsies; (4) willingness to abstain from vaginal sex for 15 days as part of the study; (5) antiretroviral treatment (ART) naïve and (6) general good health. Exclusion criteria were: (1) pregnancy; and (2) active menstruation. All HIV-uninfected female sex workers were currently active in sex work and had been enrolled in the Majengo Clinic for at least three years; thereby meeting previously published epidemiologic criteria for relative HIV resistance [3]. All lower risk individuals enrolled reported no history of sex work and only one sexual partner for the last 6 months. The detected HIV viral load in the HIV-infected women was 20–64800 copies/mL (median: 11735 copies/mL) and the CD4 count ranged 121–1737 cells/µL (median: 493 cells/µL).

### Genital Sampling at Enrolment Visit

Cervical biopsies were collected using a protocol previously shown to be safe and well-tolerated in Swedish participants at low risk of HIV exposure [12]. An external and internal genital exam was performed and cervicovaginal secretions (CVS) were collected from all women by rotating one cotton-tipped swab 360° in the cervical os, and one swab to collect secretions from the posterior vaginal fornix. Both swabs were transferred into a vial containing 5 mL of phosphate-buffered saline (PBS).Next, cervical cells were collected by rotating one cytobrush 360° in the cervical os and two ectocervical biopsies (3 mm^2^) from the superior portion of the ectocervix were collected with Schubert biopsy forceps (B. Braun Aesculap AG, Tuttlingen, Germany). A polycresulin gel was applied after sampling to induce vasoconstriction and subsequent homeostasis and all participants were observed for up to one hour to ensure that no active bleeding occurred prior to clinic discharge.

### Other Study Procedures

Data regarding demographical, reproductive, sexual and clinical characteristics were collected using a questionnaire. All participants were provided with both written and verbal information about the potential for cervical biopsy to increase the risk of HIV acquisition and/or transmission, this information was repeated at two separate clinic visits prior to enrolment. Participants were asked to abstain from unprotected vaginal sex for a period of one day prior to the procedure and to refrain from any vaginal sex, vaginal douching or tampon insertion for fourteen days after the procedure. Since the study protocol precluded income from sex work, FSW participants received monetary compensation equivalent to the expected lost income; lower risk controls received the same compensation. Genital infection diagnostics included: HIV (Chiron, Emeryville, CA, USA) and Herpes Simplex Virus type 2 (HSV2) serology (HerpeSelect® 1 and 2 Immunoblot IgG, Focus Diagnostics, CA, USA); urine for *Chlamydia trachomatis* and *Neisseria gonorrhoeae* (Amplicor PCR Diagnostics, Roche Diagnostics, Quebec, Canada); syphilis serology (Macro-Vue Rapid Plasma Reagin test, Becton Dickinson, Franklin Lakes, NJ, USA); Gram stain for bacterial vaginosis (BV; defined as a Nugent score of 7 to 10) and lactobacillus colonization and vulvovaginal candidiasis (VVC) (defined as the Gram stain finding of any lactobacilli or yeast, respectively). All participants were provided with HIV/STI prevention counseling, male and female condoms, family planning services, treatment of STIs, medical care for acute and chronic illnesses, access to adequate diagnostic testing and referral for specialist consultant and/or hospitalization at Kenyatta National Hospital if needed.

### Clinical Follow-up

Study participants were asked to return for clinical follow-up 3–5 days post-procedure; those who were actively menstruating were requested to come back as soon as menses had ceased. Participants were clinically evaluated and asked about any bleeding or discomfort, and whether they had had vaginal sex since the procedure. A gynecological exam was performed, including clinical assessment of biopsy healing. The biopsy site was considered as healed when no bleeding from biopsy site, no hyperemia and no abnormalities were detected during the macroscopic evaluation. Vaginal lavages (VagL) were collected by gently aspirating 2 mL of PBS without getting in contact with the cervix. Participants were again informed of the importance of sexual abstinence for a full two weeks post-procedure.

### Measurement of Prostate Specific Antigen (PSA)

PSA levels in enrolment CVS and follow up VagL were assayed using a Chemiluminescent microparticle immunoassay **(**ARCHITECT Instrument, Abbott Laboratories, IL, USA) as a marker of recent unprotected sex [13].

### Statistical Analysis

Univariate analyses were performed using Fisher’s exact test when comparing categorical variables, and non-parametric Mann-Whitney test when comparing continuous variables. Forward conditional binary logistic regression analyses were performed with healing status of biopsy site as the dependent variable and all variables associated with non-healing of the biopsy site in univariate analysis as covariates. P-value of <0.05 was considered significant. Software products used were Prism 5.00 (GraphPad Software Inc, CA, USA) for Windows and PASW Statistics 18 (SPSS/IBM Corporation, NY, USA).

## Results

### Participant Demographics and Clinical Data

Participants, including HESN sex workers (n = 18), HIV-infected sex workers (n = 20) and HIV seronegative lower risk individuals (low-risk; n = 21) were similar, except for HSV2 seropositivity and douching practices which was lower in the low-risk participants as compared to FSW (P<0.0001, both) (Table 1). Further, HESN individuals used more over-the-counter analgesics (P = 0.023). To avoid semen contamination of CVS, abstinence from unprotected vaginal intercourse had been requested for one day prior to the biopsy. PSA levels <1.0 ng/mL was considered an indicator that unprotected vaginal sex was less likely to have occurred within 48 hours prior to sampling [14]. Eight women reported protected vaginal intercourse within 24 hours prior to the biopsy (data not shown) and 2/59 participants tested positive for PSA; one HIV-infected FSW and one low-risk control. However, PSA may be positive for up to 48 hours after unprotected sex and only 24 hours of pre-procedure abstinence had been requested.

**Table 1:** Enrolment characteristics of study population at date of biopsy.

	Low-risk^a^(*n* = 21)Median or number(range or %)		HESN FSW^b^(*n* = 18)Median or number(range or %)		HIV+ FSW^c^(*n* = 20)Median or number(range or %)	
Age (years)	39	(24–49)	42	(27–51)	42	(24–58)
Pregnancies (including abortions)	3	(0–9)	3	(1–13)	3	(0–6)
Hormonal contraception	6	(29%)	8	(44%)	5	(26%)
Pre-ovulatory mcs^d^	4	(19%)	6	(33%)	4	(20%)
Post-ovulatory mcs	11	(52%)	6	(33%)	12	(60%)
Undefined^e^ mcs	6	(29%)	5	(28%)	4	(20%)
High^f^ douching practices	6	(29%)	15	(83%)	15	(75%)
Intermediate^g^ douching practices	4	(19%)	3	(17%)	5	(25%)
Low^h^ douching practices	11	(52%)*	0	(0%)	0	(0%)
Cervical ectopy	5	(24%)	1	(6%)	2	(10%)
Bacterial vaginosis	3	(14%)	4	(22%)	3	(15%)
Vulvovaginal candidiasis	2	(10%)	2	(11%)	1	(5%)
*Chlamydia trachomatis*	1	(5%)	0	(0%)	0	(0%)
*Neisseria gonorrhea*	0	(0%)	0	(0%)	0	(0%)
HSV2 seropositive	11	(52%)*	17	(94%)	20	(100%)
Syphilis seropositive	1	(5%)	3	(17%)	6	(30%)
Current analgesics^i^	0	(0%)	4	(22%)**	1	(5%)

a HIV seronegative lower risk individuals.

b Highly exposed HIV seronegative female sex workers.

c HIV seropositive female sex workers.

d Menstrual cycle stage.

e Subjects with amenorrhea due to long-term hormonal treatment or menopause.

f Douching performed >1 time/day with water and soap or water and salt.

g Douching performed 1/week to 1/day with water only.

h Douching never performed or <1 time/week with water only.

i Over-the-counter analgesic medications potentially containing acetylsalicylic acid or non-steroidal anti-inflammatory (NSAID) components.

* P<0.05 for comparison with HESN and HIV+ FSWs (Fisher’s exact test).

** P<0.05 for comparison with LR and HIV+ FSWs (Fisher’s exact test).

### Compliance with Post-procedure Directives

In junction with a previous study on low risk Swedish control participants [12], pilot work had shown macroscopic healing at the biopsy site after 2–3 days in a subset of participants with daily clinical follow up. Therefore 3–5 days after biopsy procedure was selected as optimal for post-procedure clinical follow up and the adherence was good (n = 50, 85%); three individuals returned too early, and six individuals delayed follow up due to the onset of menses. All participants except one lower-risk control reported post-procedure sexual abstinence at the clinical follow up visit. The one participant who self-reported recent vaginal sex had a positive PSA assay, as did one additional lower-risk control. All other (n = 57, 97%) samples were PSA negative, including all samples from female sex worker participants.

### Post-procedure Outcomes

Biopsy site healing was assessed for all participants, with complete macroscopic visual healing at clinical follow-up date in 48/59 (81%). Healing status was not associated with age, hormonal contraceptive use, stage of the menstrual cycle, cervical ectopy, douching practices, or the time interval to follow-up (Table 2). Neither the presence of assessed STI’s or BV were associated with delayed healing, except for VVC (OR 8.4, 95% CI 1.2–59). Delayed healing was also more common in the HESN group (OR 11, 95% CI 2.5–51) and in women using analgesics (OR 8.4, 95% CI 1.2–59) (Table 2). Binary conditional logistic regression was performed incorporating all variables associated with non-healing of the biopsy site in univariate analysis (P<0.05) (Table 2). Non-healing remained independently associated with HESN status (adjusted OR, 14; P<0.0001) and presence of VVC (adjusted OR, 13; P = 0.023).

**Table 2:** Characteristics of study population with or without healed biopsy site at follow-up date.

	Healed^a^Median or number(range or %)		Delayed healing^b^Median or number(range or %)	
Low-risk^c^ study group (n = 21)	19	(90.5%)	2	(9.5%)
HESN FSW^d^ study group (n = 18)	10	(56%)	8	(44%)*
HIV+ FSW^e^ study group (n = 20)	19	(95%)	1	(5%)
Interval (days)^f^	5	(2–11)	4	(2–11)
Age (years)	40	(24–58)	41	(34–47)
Pregnancies (including abortions)	3	(0–13)	3	(2–5)
Hormonal contraception (n = 19)	14	(74%)	5	(26%)
Pre-ovulatory mcs^g^ (n = 14)	11	(79%)	3	(21%)
Post-ovulatory mcs (n = 30)	26	(87%)	4	(13%)
Undefined^h^ mcs (n = 15)	11	(73%)	4	(27%)
High^i^ douching practices (n = 36)	27	(75%)	9	(25%)
Intermediate^j^ douching practices (n = 12)	10	(83%)	2	(17%)
Low^k^ douching practices (n = 11)	11	(100%)	0	(0%)
Cervical ectopy (n = 8)	7	(88%)	1	(12%)
Bacterial vaginosis (n = 10)	8	(80%)	2	(20%)
Vulvovaginal candidiasis (n = 5)	2	(4%)	3	(60%)**
*Chlamydia trachomatis* (n = 1)	1	(100%)	0	(0%)
*Neisseria gonorrhea* (n = 0)	0	(0%)	0	(0%)
HSV2 seropositive (n = 48)	37	(77%)	11	(23%)
Syphilis seropositive (n = 10)	9	(90%)	1	(10%)
Current analgesics^l^ (n = 11)	8	(73%)	3	(27%)*

a Biopsy site completely healed at follow-up.

b Biopsy site macroscopically still raw but no active bleeding at follow-up.

c HIV seronegative lower risk individuals.

d Highly exposed HIV seronegative female sex workers.

e HIV seropositive female sex workers.

f Number of days from biopsy procedure to follow-up.

g Menstrual cycle stage.

h Subjects with amenorrhea due to long-term hormonal treatment or menopause.

i Douching performed >1 time/day with water and soap or water and salt.

j Douching performed 1/week to 1/day with water only.

k Douching never performed or <1 time/week with water only.

l Over-the-counter analgesic medications potentially containing acetylsalicylic acid or non-steroidal anti-inflammatory (NSAID) components.

* P≤0.05 for comparison of delayed healing with LR and HIV+ FSWs (Fisher’s exact test).

** P≤0.05 for comparison of healed and delayed healing (Fisher’s exact test).

### HIV Status at Follow Up

Study participants who were initially HIV seronegative (lower risk controls and HESN female sex workers, n = 39) were reassessed at six months for serological HIV testing. All participants remained HIV uninfected. No gynecological exam was performed at this time.

## Discussion

Elucidating the mucosal immune correlates of HIV susceptibility and protection is an important research focus for the vaccine and microbicide field, but genital immune studies are hampered by limitations in current sampling techniques. Cervical biopsy sampling might improve research capacity, but little is known about the safety and tolerability of such sampling within HIV endemic regions, particularly within HIV high-risk populations such as female sex workers. To our knowledge we have performed the first methodological evaluation of cervical biopsy sampling within an HIV-endemic area and an HIV high-risk cohort. The ectocervical biopsy protocol was well tolerated among study participants, with no reported adverse effects beyond minor same-day bleeding and discomfort. Healing was generally rapid, and importantly no HIV acquisition was observed among initially seronegative participants. The 3–5 day period between biopsy and follow up appeared reasonable on the basis of the presented data, and was well-accepted among the participants.

Although healing was generally rapid, it was incomplete at five days for 15% of participants. Incomplete healing was not associated with HIV status, hormonal contraception, stage of menstrual cycle or presence of cervical ectopy, although those factors would be important to include as potential confounders in any immune studies. Classical STIs such as chlamydia and gonorrhea were infrequent or absent, and so their effect could not be assessed; HSV2 and syphilis seropositivity was not associated with healing. However, VVC was common and was associated with impaired healing, suggesting that screening and treatment of VVC in advance of cervical biopsy, in addition to general STI screening, might be advisable. As the specificity and sensitivity of clinical VVC diagnosis may be low we advise this diagnosis to be verified microscopically.

Further, we observed that incomplete healing was more common within HESN participants. The basis for this was not clear, but was not associated with co-infections such as HSV2 that were increased in this group. HESN individuals have recently been shown to demonstrate relative “immune quiescence”, based on low resting levels of proinflammatory cytokine expression, higher levels of T regulatory cells and down regulation of genes in key signaling pathways of HIV infection [15], [16], [17]. In theory this might contribute to impaired recruitment and activation of granulocytes and monocytes, with a subsequent delay in healing, although this will require further study. The observed association between slower healing at biopsy site and use of analgesics could possibly derive from the known inhibition of NSAID or acetylsalicylic acids on blood platelet function. Although this correlation lost statistical significance in the multivariate analysis, a clinical assessment and encouragement of restricted use (or use of paracetamol when indicated) may be appropriate.

In general, a high degree of compliance with the instructions from the clinical staff regarding the fifteen-day period of abstinence from recent (within 48 hours) unprotected vaginal sex was confirmed at the clinical follow up visit by the objective assessment of PSA levels. Although self-reported information may be limited in assessing the underlying reason for delayed healing, (neither vaginal douching, insertion of tampons or engagement in protected vaginal sex earlier than instructed would be picked up by PSA screening) 82% of participants had healed macroscopically by 5 days. Nevertheless, since delayed healing was observed in a minority of participants (15%) at five days, recommending a longer period of sexual abstinence (two weeks) will remain as the standard procedure for these studies at our site to maximize safety precautions. In order to support compliance with these recommendations, it will be important to include financial compensation at an appropriate level for female sex workers who would otherwise be without income for a substantial period. However, the finding of PSA in two lower risk women reminds us that abstinence from sexual intercourse that is not related to sex-work may be more difficult to achieve and that rigorous HIV/STI prevention counseling and the provision of male and female condoms is as important as financial compensation. In summary, ectocervical biopsy as a means to obtain genital samples for immunology studies in high-risk populations such as female sex workers from an HIV endemic area appeared to be safe and well tolerated when a high compliance with the requested period of post-procedure sexual abstinence was achieved Despite macroscopic healing by five days in most participants, supporting a more prolonged period of sexual abstinence will be important for safety reasons; promoting a restricted use of NSAID/acetylsalicylic acid components as well as pre-screening and treatment for VVC, in addition to general STI screening, should also be considered.
